# Drug induced subacute cutaneous lupus

**DOI:** 10.1093/rap/rkaf045

**Published:** 2025-04-21

**Authors:** Richard Porter, Jennifer Bendelow, Pippa Watson, Christos Tsekos, Janice Ferguson

**Affiliations:** Department of Rheumatology, Wythenshawe Hospital, Manchester Foundation Trust, Manchester, UK; Department of Rheumatology, Wythenshawe Hospital, Manchester Foundation Trust, Manchester, UK; Department of Rheumatology, Wythenshawe Hospital, Manchester Foundation Trust, Manchester, UK; Department of Rheumatology, Wythenshawe Hospital, Manchester Foundation Trust, Manchester, UK; Department of Dermatology, Wythenshawe Hospital, Manchester Foundation Trust, Manchester, UK

A 72-year-old woman with Sjögren’s disease presented with a rash that failed to respond to antifungals and topical steroids. Her medical history included hypercholesterolaemia and gastro-oesophageal reflux, with omeprazole initiated 10 months ago. Examination revealed a florid annular papulosquamous eruption occurring on the upper back and cheek (Fig. 1). Blood tests showed positive ANA and SS-A, negative dsDNA and normal complement and eosinophilia. It was felt that skin biopsy was not required for diagnosis. A clinical diagnosis of scLE was made and topical tacrolimus was initiated with limited success. Omeprazole was identified as a possible causative factor and was stopped. The rash completely resolved within 8 months. Dyspeptic symptoms were successfully treated with Ranitidine. scLE presents as a non-scarring scaly rash affecting the upper chest, arms and face. scLE is mostly an autoimmune disorder, but 20–40% of cases are drug induced. A higher proportion of these are older patients, reflecting increasing polypharmacy with age. The >100 known causative agents include proton pump inhibitors (PPIs), thiazides, terbinafine, TNF-α inhibitors and NSAIDs [1]. Patients have positive ANA, whether drug induced or not, in 80% of cases. dsDNA positivity is rare in drug-induced cases. Anti-histone antibodies are found in 8% of PPI-induced scLE [2]. Treatment involves sun protection, topical steroids, hydroxychloroquine oral steroids and removal of causative drugs [3].

**Figure 1.**
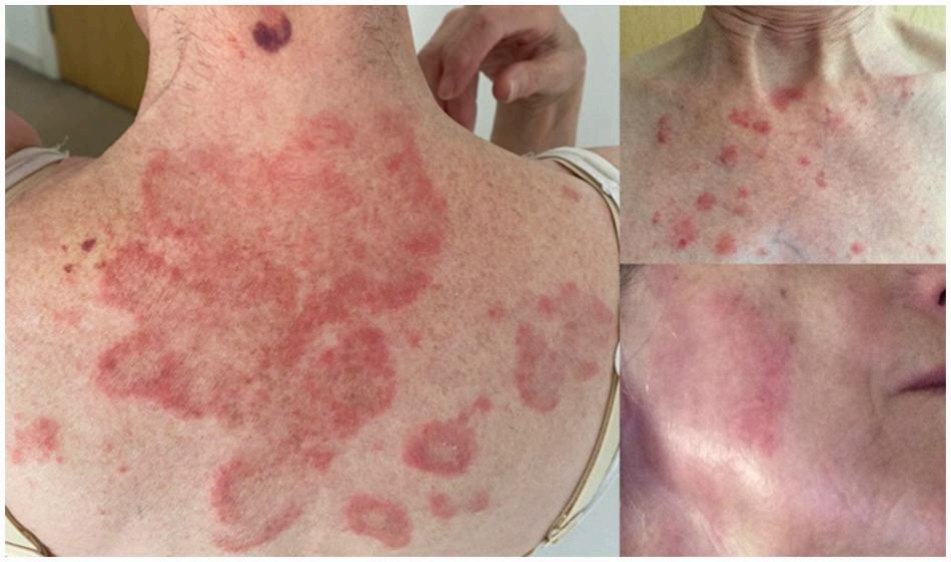
Florid annular papulosquamous eruption experienced by the patient over the upper back, chest and right cheek, which are typical in scLE

## Data Availability

The data underlying this article are available in the article.
